# Spontaneous Gallbladder Perforation: A Case Report

**DOI:** 10.7759/cureus.32249

**Published:** 2022-12-06

**Authors:** Yitong Xiao, Robert Salem, Shafaq Maula, Cassie Belanger, Frederick Tiesenga

**Affiliations:** 1 Surgery, Saint James School of Medicine, Chicago, USA; 2 Surgery, Community First Medical Center, Chicago, USA; 3 General Surgery, West Suburban Medical Center, Chicago, USA

**Keywords:** cholecystitis, cholecystectomy, cancer gallbladder, emergent general surgery, gallbladder perforation

## Abstract

Emphysematous cholecystitis (EC) is an acute infection caused by gas-forming organisms and is considered a surgical emergency. The presenting symptoms of EC are often difficult to distinguish from those of uncomplicated acute cholecystitis, necessitating the use of CT for diagnosis. EC is associated with higher rates of gangrene and perforation of the gallbladder compared to typical acute cholecystitis. It is also associated with significantly higher rates of mortality.

In this report, we discuss the case of a 57-year-old African American female who presented to the emergency room with nausea, non-bloody vomiting, and abdominal pain for three days. Physical examination showed a soft but tender abdomen, especially in the right upper quadrant, and labs showed leukocytosis of 15.5/mm^3^. A CT of the abdomen and pelvis was ordered, which demonstrated air in the gallbladder lumen with extraluminal air adjacent indicating ruptured EC.

## Introduction

Emphysematous cholecystitis (EC) is a rare complication of acute cholecystitis characterized by the formation of gas inside the lumen or wall of the gallbladder. Due to the high mortality rates associated with perforated EC, it is considered a surgical emergency. Compared to acute cholecystitis, male patients are twice as likely to develop a perforated EC than their female counterparts, with the majority of patients having comorbidities such as diabetes mellitus [1]. Physical examinations such as Murphy's sign that include tenderness in the upper right quadrant can raise suspicion for acute cholecystitis. However, a CT scan of the abdomen is considered the most sensitive and specific diagnostic tool to identify gas in the lumen or wall of the gallbladder, with the presence of pneumoperitoneum being diagnostically significant for perforation [2].

## Case presentation

A 57-year-old African American female patient presented to the emergency room complaining of right upper quadrant abdominal pain, nausea, and non-bloody vomiting for three days. The patient stated that she had tacos three nights ago, and she believed that her symptoms were food poisoning-related. Her abdominal pain had been getting worse, and she denied any aggravating or relieving factors related to her pain. The patient stated that the abdominal pain was generalized but was more prominent in the upper abdominal region. She denied diarrhea or chills. The patient had tried bismuth subsalicylate for symptom relief but it had been ineffective. She denied any travel history or contact with sick individuals.

**Figure 1:**
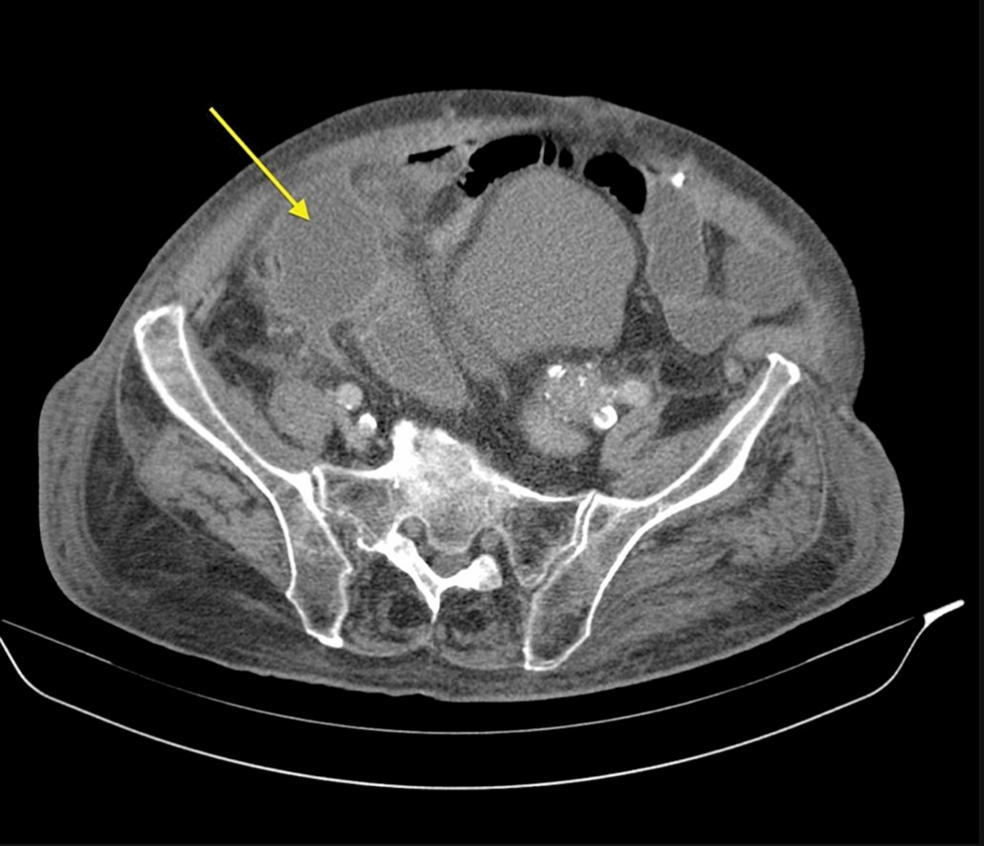
CT scan showing perforated gallbladder (arrow) CT: computed tomography

The patient’s past medical history was unremarkable; she had undergone abdominoplasty and mastectomy 20 years ago, but no other abdominal surgeries. The patient denied any history of substance abuse, smoking, and alcohol abuse. She was assessed at the bedside, which revealed moderate distress secondary to pain, nontoxic, slightly tachycardic but no murmurs or gallops. Her abdomen was soft but tender to palpation specifically in the right upper quadrant and epigastric region. A positive Murphy's sign was present with a negative McBurney’s point tenderness and there was no distention or guarding rebound. Laboratory tests obtained at triage demonstrated leukocytosis of 15.5/mm^3^. Abdominal and pelvic CT with contrast indicated air in the gallbladder lumen with adjacent extraluminal air, compatible with ruptured EC (Figure 1). Imaging studies could not exclude a fistula between the gallbladder and duodenum.

**Figure 2:**
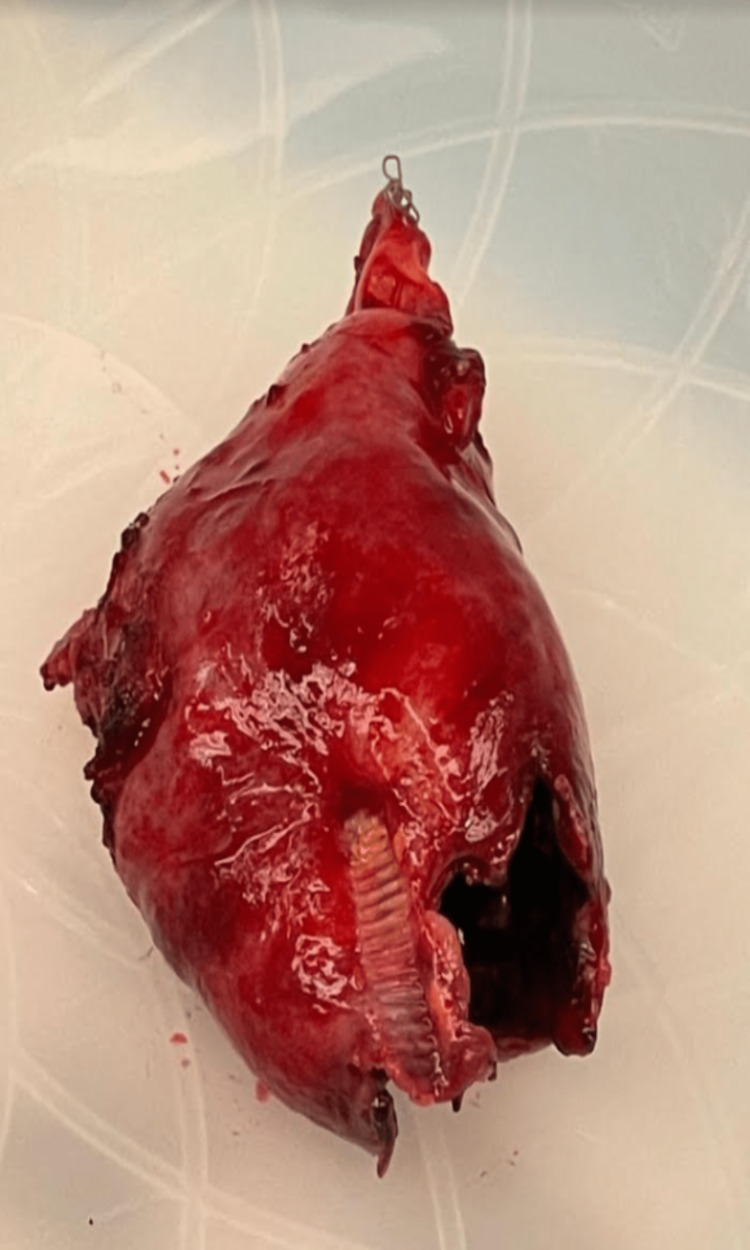
The perforated gallbladder

The patient was given Zosyn and the general surgery department was consulted immediately. Given the patient's deteriorating clinical condition and severe pathological indications on imaging studies, she was taken to the operating room immediately and prepped and draped after the induction of general anesthesia. At this time, a generous right upper quadrant Kocher incision was made. The peritoneal cavity was entered. There was a massive amount of inflammation in the right upper quadrant. The omentum was tightly adherent to the liver. After dissecting and getting access to the liver, the gallbladder was identified and found to be freely ruptured into the omentum and necrotic in appearance. The gallbladder was slowly, methodically, and painstakingly dissected out in a retrograde fashion with cautery (Figure 2). The cystic duct and artery area were identified and they were fairly inflamed as well. Utilizing a complete dome-down technique and showing utmost care to stay away from the critical structure, Endo GIA™ was employed to access the cystic duct and artery without event. Antibiotic irrigation was utilized and retrieved. Jackson-Pratt (JP) drain was left in the abdomen and brought out through a separate stab incision. The specimen was sent to pathology, and cultures were taken. The wound was closed anatomically, and the procedure was completed.

The patient was monitored in the ICU for four days postoperatively. After transferring to the floor, it was found that the patient's liver transaminase was trending up. A hepatobiliary iminodiacetic acid (HIDA) scan was ordered to rule out biliary tract leakage. After consulting with the multidisciplinary care team, the primary care department and infectious disease department believed that the patient was having transaminitis. Viral hepatitis was considered unlikely since the hepatic viral panel was negative, but it was felt that the etiology could be due to antibiotics and acetaminophen. After nearly a week of medical management, the patient was discharged on postoperative day seven. She was seen in the office one week postoperatively and was doing fine with a nicely healed incision site. Her biopsy results were negative for any malignancy, which was communicated to her.

## Discussion

Cholecystitis with or without cholelithiasis is the most common cause of gallbladder perforation. In elderly patients, spontaneous gallbladder perforation can result from a decrease in its blood supply, which can be due to atherosclerosis, focal vasospasm, or localized vasculitis [3]. Acute biliary emergencies that involve the gallbladder make up around 2% of cases, and due to the high mortality and morbidity rates associated with them, they pose a challenge in terms of treatment [4].

Niemeier [5] established a classification system for gallbladder perforation in 1934: type 1 involves an acute free perforation into the peritoneal cavity, type 2 is a subacute perforation with a pericholecystic abscess, and type 3 is a chronic perforation with a cholecystoenteric fistula. Roslyn et al. [6] have noted in their study that type 1 and type 2 gallbladder perforations are most common in young individuals aged less than 50 years, whereas type 3 is more common in the elderly population with a history of stone disease. They also developed a classification scheme for gallbladder perforation comprising three categories: spontaneous, traumatic, and iatrogenic. Acute inflammation, infection, lithiasis, congenital blockage, and anticoagulant treatment-induced are all examples of spontaneous diseases [3].

The most likely mechanism of gallbladder perforation in acute cholecystitis is cystic duct obstruction (usually caused by a stone at the neck), which causes intraluminal secretions to be retained, resulting in an increase in intraluminal pressure. This increased intraluminal pressure impairs the gallbladder's lymphatic and venous outflow, which ultimately leads to gallbladder rupture and necrosis [3]. The fact that 60% of instances of gallbladder perforation in Derici et al.’s study [7] occurred near the fundus, where there is the least blood flow, supports the significance of the ischemia process. Gallbladder perforation is a risk factor that can be increased by conditions including cholelithiasis, infections, malignancies, diabetes, atherosclerosis, and steroid therapy, among others [3].

Since there are no conventional symptoms and indicators of gallbladder perforation, clinical identification can be challenging and sometimes delayed or overlooked. According to Pedrosa et al. [8], gallbladder perforation indications can be classified as direct or indirect: the presence of calculi outside the gallbladder or a ruptured portion of the gallbladder wall is a direct indicator, while the indirect signs include the existence of an abscess outside the gallbladder and gallstones, as well as gallbladder wall thickening. The sensitivity of CT scans in detecting gallbladder perforation and biliary calculi has been found to be between 88% and 89% [9]. MRI may reveal the gallbladder wall as well as abnormalities. In suspected cases of acute cholecystitis, magnetic resonance cholangiopancreatography (MRCP) shows the biliary tree better than other modalities in the context of gallbladder perforation [10].

## Conclusions

Gallbladder perforation is a rare and emergent medical condition that indicates acute surgical care based on imaging modalities such as a CT scan. Early diagnosis and an emergent cholecystectomy can considerably reduce morbidity and mortality, as in our case. The clinical encounter of a ruptured gallbladder always warrants an aggressive investigation to rule out etiologies such as malignancy.
